# The Mechanobiology of Adipocyte Hypertrophy: Cytoskeletal Remodelling as a Driver of Insulin Resistance in Obesity

**DOI:** 10.1002/cbf.70283

**Published:** 2026-08-12

**Authors:** Dhakshmi Sasankan, Renu Mohan

**Affiliations:** ^1^ Department of Biotechnology University of Kerala, Kariavattom Campus Thiruvananthapuram India

**Keywords:** adipocyte hypertrophy, cytoskeleton, insulin resistance, mechanotransduction, septin, YAP/TAZ

## Abstract

Adipocyte hypertrophy poses an important mechanical challenge—adipocytes can increase in volume up to a thousand‐fold while still maintaining their structural integrity. Although lipid metabolism is well studied, little is known about the cytoskeletal adaptations that are required to accommodate this expansion and the resulting pathological consequences. This review critically examines how the cytoskeletal remodelling is associated with insulin resistance. We explore how mechanotransduction pathways like YAP/TAZ, which inhibit the expression of adipogenic genes, are triggered when cortical actin transforms into stress fibres. This review also discusses the role of the microtubule network in lipid droplet fusion and the vimentin cage that regulates lipolysis. Further, we highlight the emerging role of the Septin family (SEPT), specifically the conflicting pro‐ and anti‐adipogenic roles of SEPT7, where SEPTs might act as a molecular brake on adipocyte expansion. We conclude that obesity‐induced insulin resistance is partially due to a failure of cytoskeletal mechanics, making cytoskeletal regulators potential therapeutic targets for metabolic disease.

## Summary

Adipocytes face a unique physical challenge: maintaining the cellular integrity even when they are expanding their volume up to a thousand‐fold. This review elucidates how the physical expansion is related to metabolic dysfunction by integrating the roles of actin, microtubules, vimentin and SEPTs. It also describes how the structural support mechanisms, such as microtubule stabilization and actin‐mediated tension, actively impede insulin signalling by sequestering GLUT4 vesicles and triggering the Piezo1‐Actin‐YAP/TAZ axis. By reframing obesity‐induced insulin resistance as a consequence of cytoskeletal mechanics, this review identifies the cytoskeleton not merely as a scaffold, but as a potential therapeutic target for metabolic disorders.

## 1 Introduction

Obesity is a global health crisis, with the World Health Organization estimating that over 40% of the adult population is overweight and more than 16% are living with obesity. The consequences of obesity, such as cardiovascular disease, type 2 diabetes, non‐alcoholic fatty liver disease and chronic inflammation, are well characterized, but the aetiology of these disorders starts at the cellular level within the adipose tissue [1]. The expansion of adipose tissue occurs through two main mechanisms: adipocyte hyperplasia (creating new adipocytes from progenitor cells) and adipocyte hypertrophy (the enlargement of existing adipocytes). Unlike hyperplasia, hypertrophy puts significant structural and functional stress on the mature adipocytes when nutrient storage exceeds their physiological limits [2, 3]. The initial, moderate hypertrophic expansion represents a healthy adaptive response to positive energy balance, but chronic or excessive lipid over‐loading leads to pathological over‐expansion, and it is the main cause of metabolic dysfunction. As the cell volume expands, it induces chronic cellular stress characterized by mitochondrial impairment, endoplasmic reticulum stress, and mechanical strain on the plasma membrane [4, 5, 6]. Real‐time biophysical measurements in mouse 3T3‐L1‐derived adipocytes have shown that the mechanical properties of differentiated adipocytes depend on their level of maturation and the stiffness increases significantly due to the intracellular lipid droplet accumulation [7]. The lipid‐overloaded hypertrophic cells also exhibit other critical abnormalities such as accumulation of calcium and cholesterol crystals, which trigger cell death via pro‐inflammatory pyroptosis [8]. The physical expansion of adipocytes correlates directly with the onset of systemic insulin resistance [9, 10]. In adult obesity, hypertrophy is the main process promoting adipose tissue growth, especially in visceral areas [11, 12].

The excessive enlargement of adipocytes creates a hypoxic tissue environment due to increased distances for oxygen diffusion. This localized hypoxia stabilizes Hypoxia‐Inducible Factor 1α (HIF‐1α), which initiates a pro‐fibrotic transcriptional programme that alters the extracellular matrix (ECM) composition [13]. The resulting dense ECM network rigidifies the tissue microenvironment, creating a pathologically stiff phenotype that encapsulates expanding adipocytes and limits further healthy expansion [14, 15]. Hypertrophic adipocytes increase the secretion of adipokines and cytokines, which require adaptations in the secretory pathways. Hypertrophic adipocytes also increase the secretion of pro‐inflammatory cytokines such as TNF‐α and IL‐6, which results in chronic low‐grade inflammation and correlates directly with increasing adipocyte diameter [16]. When adipocytes reach their maximum storage capacity, the metabolic balance shifts toward a state of chronic fatty acid efflux. This is due to an increase in basal lipolysis and failure of insulin to suppress fatty acid release via the PDE3B/cAMP pathway due to insulin resistance. In healthy adipocytes, insulin suppresses lipolysis by promoting the activation of phosphodiesterase 3B (PDE3B), an enzyme that degrades cyclic AMP (cAMP) [17, 18, 19]. When cAMP levels are decreased, it deactivates hormone‐sensitive lipase, preventing the breakdown of stored triglycerides. When the adipocytes become insulin resistant, insulin will not be able to stimulate PDE3B and cAMP levels increase. The increased accumulation of cAMP activates Protein Kinase A, which directly phosphorylates HSL and indirectly, via perilipin, activates adipocyte triglyceride lipase (ATGL), causing the enzymatic hydrolysis of triglycerides and the release of free fatty acids into the bloodstream [20, 21]. This results in the accumulation of ectopic fat in organs such as the liver, heart, and muscles. Adipocyte dysfunction together with ectopic lipid deposition causes inflammation and impairs systemic glucose metabolism, resulting in systemic insulin resistance characteristic of lipotoxicity [22, 23].

Despite making remarkable progress in understanding the metabolic and signalling pathways that regulate adipocyte function, obesity remains a complex, chronic and relapsing disease for which there is currently no simple curative therapy. The current medications, such as agonists of receptors for glucagon‐like peptide‐1 (GLP‐1), glucose‐dependent insulinotropic peptide (GIP) and glucagon, target nutrient‐stimulated hormonal pathways to effectively control appetite and body weight [24, 25, 26]. However, they function as long‐term management tools for obesity rather than as permanent cures because when these treatments are stopped, it often leads to metabolic adaptation and weight gain. The weight gain happens because, following weight loss, the body induces hormonal changes (such as increased ghrelin, decreased leptin) and a reduction in resting metabolic rate, which predisposes individuals to weight regain and relapse of metabolic symptoms [27, 28, 29]. Furthermore, these treatments do not directly address the structural and mechanical dysfunction of the adipocytes associated with obesity.

Although the metabolic pathways in obesity are extensively studied, the physical and mechanical challenges associated with adipocyte hypertrophy are often overlooked. In a mature white adipocyte, the nucleus and cytoplasm are compressed into a thin cortical rim to create space for a large unilocular lipid droplet that can take up to more than 95% of the cell volume. This creates a mechanical challenge to the cell, where it must be flexible enough to expand its volume up to a thousand times, but at the same time, it must maintain homoeostasis and structural integrity to prevent cellular dysfunction [30]. In order to support these changes and avoid mechanical failure during extreme hypertrophy, the cytoskeleton, which is the dynamic network of actin filaments, microtubules, intermediate filaments and SEPTs, undergoes extensive remodelling.

To develop therapies for obesity and obesity related diabetes, we must look beyond the metabolic enzymes and understand the cellular machinery that governs adipocyte expansion. The cytoskeleton is often considered a passive structural scaffold. Here, we discuss the recent data which shows that the cytoskeleton functions mainly as a mechanosensor, converting the physical strain of lipid droplet expansion into biochemical signals that control the cell fate. By connecting cytoskeletal dynamics to metabolic regulation, this review tries to identify specific targets to dissociate insulin resistance from obesity, so that adipose tissue can expand without triggering the associated metabolic disorders. To provide a cohesive framework for this review, we propose that adipocyte hypertrophy could be viewed as a sequential mechanical failure. First, the cell must sense the physical stretch (Actin); second, it must maintain intracellular transport despite physical compression (Microtubules); third, it must preserve its mechanical and structural integrity during expansion (Vimentin); and finally, it must use molecular brakes to prevent pathological over‐expansion (SEPTs). By organizing these processes as a coordinated response to mechanical stress, we can better understand how cytoskeletal dysfunction serves as the link between physical obesity and metabolic insulin resistance.

## 2 The Actin Cytoskeleton: From Docking Station to Mechanosensor

In adipocytes, actin filaments generate the tension necessary to maintain adipocyte structure. Traditionally, they were considered merely as tracks for GLUT4 vesicle transport, and the cortical actin network works along with the plasma membrane to resist structural strain during cell expansion. Recent evidence reveals that the actin network, together with mechanosensitive ion channels like Piezo1, forms a primary mechanosensor of the cell, converting the physical strain of hypertrophy into transcriptional programmes that regulate adipose tissue remodelling. Pathological overactivation of this mechanosensitive apparatus during severe expansion triggers chronic inflammatory pathways, ultimately driving insulin resistance [31, 32, 33]. To understand how mechanical forces regulate adipocyte physiology, we must clearly distinguish between the intrinsic strain exerted by the expanding lipid droplet and the extrinsic constraints imposed by the tissue microenvironment.

### 2.1 Intrinsic Forces: Lipid Droplet Expansion and Cortical Strain

Before looking into the pathological hypertrophy, it is important to understand the cytoskeletal dynamics required for healthy adipogenesis. Pre‐adipocytes have a fibroblast‐like shape with a dense network of actin stress fibres. In order to differentiate into a mature adipocyte with a spherical shape, the cell reorganizes the dense actin network into a more cortical arrangement to accommodate the large unilocular lipid droplet. This remodelling is a tightly regulated process involving a significant decrease in actin gene expression [34].

The disruption of actin stress fibres is required for the induction of the master adipogenic gene PPARγ. In mouse pre‐adipocytes, high RhoA‐ROCK signalling activity maintains actin stress fibres to suppress adipogenesis [35], which promotes the nuclear translocation of the transcriptional co‐activator MKL1 (also known as MRTF‐A) to act as a repressor of PPARγ. The induction of differentiation causes downregulation of RhoA‐ROCK signalling, leading to an increase in monomeric G‐actin. The G‐actin sequesters MKL1 in the cytoplasm, preventing its nuclear translocation and thereby relieving the repression of PPARγ [36]. The disassembly of actin stress fibres and the subsequent formation of a cortical actin structure are essential for terminal differentiation, and this process is regulated by the Insulin‐PI3K‐Rac1 signalling pathway [37] and integrin‐mediated extracellular matrix interactions [38].

Actin remodelling during the adipocyte differentiation process is modulated by various actin‐binding proteins. During the differentiation of human stromal cells to adipocytes, actin filaments undergo depolymerization and remodelling, and the depolymerization is regulated by Cofilin‐1, an actin‐depolymerizing factor [39]. Another actin‐binding protein, Cotl1 (Coactosin‐like F‐actin binding protein), was also found to be essential for adipogenesis. When Cotl1 was deleted or silenced in mice or in pre‐adipocyte cell lines, there was a reduction in lipid accumulation and inhibition of cell differentiation into mature fat cells. Cotl1‐deficient mice (Cotl1 − /− mice) also showed resistance to high‐fat diet‐induced weight gain, reduced adipocyte enlargement and fat accumulation in the liver. These mice also showed improved glucose tolerance and insulin sensitivity compared to wild‐type mice, indicating protection against associated metabolic disorders. The connection between actin dynamics and metabolic gene regulation was further emphasised by the finding that Cotl1 controls the expression of the key adipogenic PPARγ gene [40].

In the mature, insulin‐sensitive adipocyte, filamentous actin (F‐actin) forms a dense cortical network immediately beneath the plasma membrane. The cortical actin helps in the docking and fusion of the GLUT4 vesicles with the plasma membrane, allowing glucose to enter the cell [41]. In spite of its critical role, there are conflicting reports on the remodelling of the actin network during adipocyte hypertrophy. Hansson et al. [42] reported a significant increase in total cellular F‐actin in hypertrophic mouse adipocytes. The increase in F‐actin is linked to increased Rho‐kinase activity and changes in the expression and phosphorylation of actin‐regulating proteins that promote polymerization [43].

These changes in actin polymerization dynamics also link the cell's structure to its metabolic function. In primary human adipocytes and hypertrophic mouse adipocyte cells, the polymerization of G‐actin into rigid F‐actin stress fibres depletes the amount of monomeric G‐actin in the cytoplasm. This causes the release of MKL1/MRTF‐A from its cytoplasmic anchor, which then translocates into the nucleus. In the nucleus, MKL1 suppresses the expression of PPARγ and its downstream target genes associated with adipogenesis, insulin signal transduction and glucose transport [36, 44]. This increase in F‐actin is a structural change which helps to accommodate the larger size of the cell. The increase in cell size is also linked to impaired insulin signalling and insulin‐stimulated glucose transport. Importantly, when mice fed on a high‐fat diet were switched to a normal diet, the adipocyte size, insulin response and F‐actin levels were all restored to normal levels, which implies that the diet‐induced changes are reversible [42].

In contrast, Kim et al. [45] reported a decrease in F‐actin and an increase in globular (G)‐actin in the cortical region of the adipocyte. During hypertrophy, when adipocytes develop a single, large unilocular lipid droplet, the cell loses its ability to respond properly to insulin. The hypertrophic adipocytes with large and unilocular lipid droplets showed defects in GLUT4 trafficking to the plasma membrane, resulting in impaired insulin‐dependent glucose uptake. The study concluded that the disintegration of the cortical actin network and changes in the F‐actin to G‐actin ratio inhibit proper trafficking and docking of GLUT4 vesicles, preventing glucose uptake, which results in insulin resistance.

Although the findings of the two studies appear contradictory, we propose that they represent the spatial redistribution of the actin cytoskeleton during adipocyte expansion. The Hansson et al. [42] study observed an overall increase in F‐actin throughout the adipocyte cell. We propose that the cell compensates for the large mechanical strain produced during hypertrophy by polymerizing actin into rigid stress fibres. The rigid stress fibres provide the structural support necessary to accommodate the expanded lipid droplet and the increased cell volume. In contrast, the Kim et al. [45] study focused on the localized reduction of F‐actin at the cell cortex when the lipid droplet expands. This decrease in cortical F‐actin at the cell cortex inhibits the trafficking of glucose transporters, leading to insulin resistance. Therefore, the above two studies show that during hypertrophy, even though the total F‐actin mass of the cell increases, the F‐actin network at the cell cortex is disrupted, which is the direct cause of metabolic dysfunction.

### 2.2 Extrinsic Constraints: The Extracellular Matrix and Mechanical Confinement

The mechanical stress associated with adipocyte hypertrophy arises not only from the internal pressure of lipid droplet accumulation but is also due to the external mechanical constraints exerted by the surrounding extracellular matrix (ECM). Collagen VI is a key ECM component whose expression is increased in obesity and directly correlates with human insulin resistance. It forms a cage around individual adipocytes, which restricts the outward expansion of the cells [14]. The cellular constraint is amplified by a dense interstitial network of other collagens such as Collagens I and III. As mentioned before in the introduction, localized hypoxia stabilizes HIF‐1α, which initiates a pro‐fibrotic programme, increasing the expression of fibrillar collagens and Lysyl Oxidase (LOX) [13]. Obesity‐associated downregulation of AMPK enhances TGF‐β signalling and LOX expression, significantly accelerating collagen cross‐linking [46]. This dense, cross‐linked ECM network rigidifies the tissue microenvironment, establishing a pathologically stiff phenotype that encapsulates expanding adipocytes [14]. The extrinsic mechanical confinement exerts forces that are distinct from the internal pressure exerted by the enlarging lipid droplet. The internal lipid accumulation creates tensile stress on the plasma membrane, whereas the external ECM rigidity activates specific integrin‐mediated mechanotransduction pathways, focal adhesion kinase (FAK) signalling and mechanosensitive ion channels. The physiological consequence of this external resistance was studied in Collagen VI‐null (col6KO) mice. In the absence of the collagen microfibrillar cage, adipocytes expanded into giant cells without experiencing severe mechanical stress. The stress‐free expansion allowed the collagen VI‐null (col6KO) mice to remain metabolically healthy and insulin sensitive in spite of having a high‐fat diet. This indicates that the pathological accumulation of Collagen VI is a critical factor involved in the mechanical and metabolic failure seen in obesity [14]. Therefore, the physical confinement imposed by a rigid ECM along with the cell expansion by lipid accumulation is a primary factor of adipocyte cellular stress, subsequent cell death, and chronic low‐grade inflammation.

### 2.3 Mechanotransduction: The Piezo1‐Actin‐YAP/TAZ Axis

The external pressure by the ECM forces intracellular cytoskeletal remodelling as the adipocyte attempts to sense and adapt to its rigid environment. Central to this remodelling is the formation of actin stress fibres, which are not only a structural adaptation that is required to accommodate the increased volume of lipid droplets, but are also a critical signalling event that regulates cellular fate [47]. Recent studies identify the mechanosensitive ion channel Piezo1 as the primary sensor triggering the hypertrophic changes in adipocytes. Piezo1 acts as the primary upstream sensor that initiates the cytoskeletal remodelling and YAP/TAZ nuclear translocation. When the cell membrane stretches due to adipocyte expansion, Piezo1 channels open to allow the influx of calcium (Ca^2+)^ ions [32]. The high intracellular calcium activates the RhoA/ROCK pathway, which induces actin polymerization and an increase in actomyosin contractility, thereby increasing intracellular tension. The cytoskeletal tension is not just confined to the cytoplasm, but it is also transmitted to the nucleus. This force causes the opening of the nuclear pores and facilitates the nuclear translocation of mechanosensitive transcriptional regulators [48].

Studies show that the Hippo signalling pathway is the key molecular link between the cytoskeletal tension and the maintenance of the mature adipocyte phenotype. The effectors of the Hippo pathway ‐ YAP (Yes‐associated protein) and TAZ (transcriptional co‐activator with PDZ‐binding motif) act as the primary sensors of cellular stiffness in metabolic tissues. The increased stiffness of the actin cytoskeleton in hypertrophic adipocytes prevents the phosphorylation of the transcriptional co‐activators YAP and TAZ [48]. The unphosphorylated YAP/TAZ moves from the cytoplasm into the nucleus. The physical connection between the actin cytoskeleton and the nucleus helps the YAP/TAZ to reach the nucleus. Further, the tension exerted by the stiff actin on the nuclear envelope causes the nuclear pores to widen, thus accelerating YAP/TAZ entry [49]. Inside the nucleus, TAZ acts as a direct transcriptional co‐repressor of PPARγ, the master regulator of adipogenesis, whereas YAP does not bind directly to PPARγ [50]. YAP suppresses adipogenesis by binding to TEAD transcription factors to induce the expression of downstream genes that signal the cell to proliferate rather than differentiate into adipocytes [48]. This critical role of YAP/TAZ in adipogenesis is further confirmed by recent findings, which show that YAP/TAZ activation represses PPARγ and can cause a reduction in fat mass by dedifferentiating mature adipocytes into progenitor‐like cells [51]. The suppression of PPARγ is usually associated with insulin resistance. By binding to the ligand‐binding domain, TAZ prevents PPARγ from recruiting co‐activators, thereby suppressing insulin‐sensitizing genes [52]. Adipocyte hypertrophy increases actin cytoskeletal tension that promotes nuclear accumulation of YAP/TAZ, which then represses adipogenic and insulin‐sensitising genes.

Even though TAZ is usually considered a suppressor of adipogenesis, its function is context‐dependent. A recent study carried out using goat adipose‐derived stem cells (gADSCs) by Zhou et al. [53] challenges the classical anti‐adipogenic function of TAZ. The study found that TAZ expression increases during early stages of adipogenic differentiation, most likely before hypertrophy occurs. Rather than inhibiting the master regulator PPARγ, TAZ activation promoted the phosphorylation of AKT and activated the PI3K/AKT signalling pathway. By activating the PI3K/AKT signalling pathway, which drives insulin signalling and cell growth, TAZ overexpression actively promotes adipogenesis of gADSCs, revealing a stage‐specific positive regulatory role for TAZ in fat cell development.

**Figure 1:**
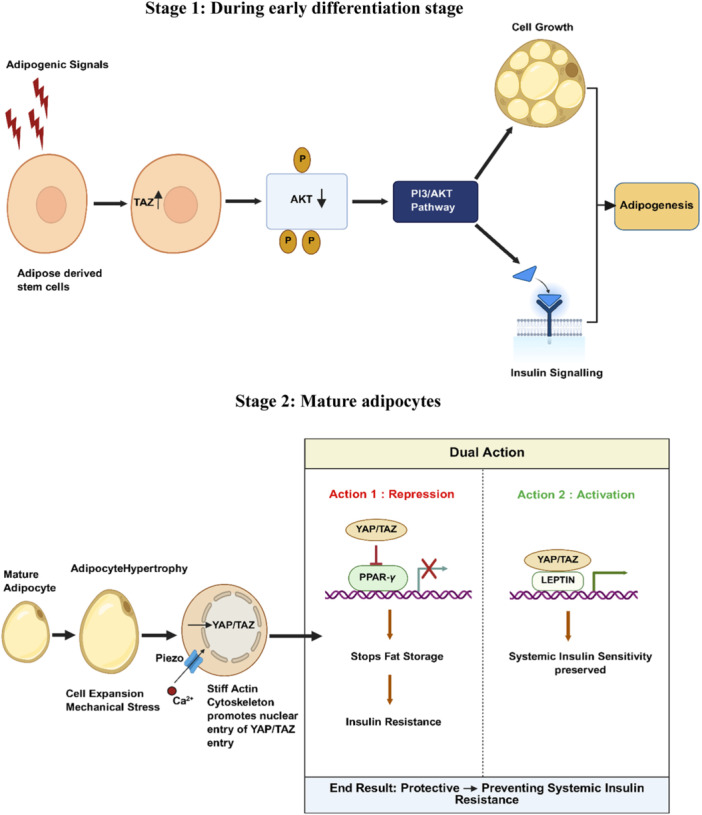
Stage‐specific regulation of adipogenesis and metabolism by YAP/TAZ Signalling. Stage 1: During the early differentiation stage, transient multilocular lipid droplets begin to form within differentiating preadipocytes. At this phase, TAZ expression increases and promotes the phosphorylation of AKT, activating the PI3K/AKT signalling pathway. This signalling promotes cell growth and enhances insulin sensitivity, facilitating the transition toward terminal adipogenesis. The multilocular morphology depicted here represents the ‘Establishment Phase’ of white adipocytes occurring prior to their coalescence into a single unilocular droplet and the onset of hypertrophy‐induced mechanical strain. Stage 2: As the mature adipocytes undergo hypertrophic expansion, there is an increase in mechanical stress, which activates the mechanosensitive ion channel Piezo1, triggering an influx of calcium ions. The high intracellular calcium induces the formation of stiff actin fibres, which promotes the nuclear translocation of YAP/TAZ. In the nucleus, YAP/TAZ exerts a dual action to manage the metabolic consequences of hypertrophy: Action 1 (Repression): YAP/TAZ suppresses the master regulator PPARγ, thereby halting further fat storage and contributing to local insulin resistance. Action 2 (Activation): Simultaneously, YAP/TAZ activates the Leptin gene. Elevated leptin levels promote systemic energy release and lipolysis, serving as a protective rescue mechanism that helps to preserve systemic insulin sensitivity in spite of localized adipocyte dysfunction. The end result of this mechanosensitive axis is a protective adaptation that tries to prevent systemic metabolic collapse during advanced obesity. Created in BioRender. https://BioRender.com/slw84ic;https://biorender.com/t5r0ua4, Stage 1: During early differentiation stage. Stage 2: Mature adipocytes.

In contrast, in a mature adipocyte experiencing severe mechanical stress (due to hypertrophy and cytoskeletal stiffness), YAP/TAZ reduces the ability of adipocytes to store fat by repressing PPARγ. The study by Choi et al. [51] in mouse models explains why this mechanical stress does not result in metabolic disorders. Normally, leptin levels decrease when fat mass decreases. However, this study found that in addition to increasing lipid storage, YAP/TAZ can also activate the leptin gene. The high levels of leptin promote energy release and lipolysis, thus preventing metabolic disorders such as diabetes. This protective mechanism by YAP/TAZ uncouples Leptin expression from fat mass, which ensures that even when the adipocyte loses storage function due to mechanical stress, high leptin levels are maintained to preserve systemic insulin sensitivity. Thus, the cytoskeleton‐signalling interaction alters cellular properties depending on the developmental stage of the adipocyte. During early adipocyte development, this interaction allows TAZ‐mediated AKT signalling to promote growth, whereas in hypertrophic mature adipocytes, cellular tension shifts the axis toward PPARγ repression and insulin resistance, which is counter‐balanced by a tension‐induced leptin rescue mechanism (Figure 1).

The function of the upstream mechanosensor Piezo1 is also context‐dependent. In mice visceral adipose tissue, Piezo1 acts as a pro‐adipogenic factor, where the mechanical stretching of Piezo1 triggers the release of Fibroblast Growth Factor 1 (FGF1). FGF1 signalling acts via FGFR1 to drive precursor differentiation and promote lipid storage [32]. But in Perivascular Adipose Tissue (PVAT) and orbital fibroblasts, Piezo1 stimulation has an anti‐adipogenic effect, where it helps to maintain cells in a fibroblast‐like state and represses key adipogenic markers such as PPARγ [54, 55]. In addition to differentiation, Piezo1 also acts as a critical sensor of adipocyte hypertrophy in established obesity. As mature adipocytes expand, the resulting mechanical stretch activates Piezo1, which initiates a pathological response that is similar to the stretch‐induced RhoA vicious cycle described by Hara et al. [56]. This Piezo1 activation drives the secretion of pro‐inflammatory cytokines such as IL‐6 and TNF‐α [33]. These cytokines act as endocrine disruptors, impairing insulin signalling in peripheral tissues and causing systemic insulin resistance. In humans with obesity and Type 2 diabetes, Piezo1 levels are significantly elevated in visceral fat and correlate directly with these inflammatory markers, confirming that the piezo channel converts the mechanical stress of obesity into metabolic dysfunctions [31].

### 2.4 Therapeutic Implications of Actin Remodelling in Adipocytes

Studies indicate that Rosiglitazone, which is an anti‐diabetic drug, targets the actin cytoskeleton to facilitate insulin signalling, and this mechanism is different from its classical nuclear receptor activity. In hypertrophic adipocytes, the cortical actin network becomes stiff and forms a dense physical barrier that prevents the translocation of GLUT4 vesicles to the cell surface. Rosiglitazone increases actin dynamics and intracellular levels of actin‐regulatory proteins, which reduces the stiffness of the cortical actin network and releases cytoskeletal tension [57]. The reorganization of the actin network helps in the fusion of GLUT4 vesicles at the plasma membrane, leading to insulin‐stimulated glucose uptake. This shows that actin dynamics can be used as a therapeutic target to reverse insulin resistance.

In addition to pharmacological agents, structural proteins can also play a protective role. Murakami et al. [58] identified a new mechanism by which obesity and insulin resistance can be prevented by modulating the expression of adipocyte adhesion molecule (ACAM), which was found to affect actin polymerization in adipocytes. Mice overexpressing ACAM were protected from high‐fat, high‐sucrose diet‐induced obesity and diabetes. Compared to the control mice, the ACAM overexpressing mice had significantly less body fat, smaller adipocytes, and better insulin sensitivity. ACAM‐mediated cell adhesion caused an increase in actin polymerization within the adipocytes. The authors hypothesized that the ACAM‐mediated F‐actin polymerization produced cytoskeletal rigidity that prevented the adipocytes from swelling and becoming excessively large, keeping them in a smaller, metabolically healthy state and preventing the pathological hypertrophy and dysfunction that result in metabolic diseases like diabetes.

Targeting the actin cytoskeleton is important for both the cell membrane and the nucleus. Reorganization of the actin cytoskeleton is not only required for altering the cell shape, but it can also affect the structure of the nucleus and nucleoli. When lipid droplets expand, they occupy the majority of the adipocyte volume and compress the nucleus against the plasma membrane. A direct, mechanically controlled relationship between the actin network and sub‐nuclear structures is suggested by the observation that actin cytoskeleton rearrangement occurs before nucleolar remodelling during adipogenesis [59]. Therefore, therapies that modulate actin tension might also relieve the mechanical stress on the nucleus. All these studies demonstrate that the actin cytoskeleton has a critical role in both normal adipocyte differentiation and pathological hypertrophy. Future therapeutic strategies targeting the RhoA‐actin‐YAP axis might reduce the cortical stiffness by modulating the cytoskeletal tension. This therapeutic strategy might ensure structural integrity to prevent nuclear stress and also enhance GLUT4 trafficking, thereby restoring metabolic health.

## 3 Microtubules: Trafficking Highway and Mechanosignalling Hub

Actin cytoskeleton controls the activities of adipocytes at the cell cortex, whereas the microtubule network regulates activities deep in the cytoplasm. This network is important for the proper function of adipocytes, because it influences differentiation, hypertrophy, lipid droplet dynamics and insulin sensitivity.

### 3.1 The Trafficking Highway: GLUT4 Transport and the Traffic Jam

In the hypertrophic adipocyte, the microtubule network faces two challenges: it has to maintain the long‐range transport of insulin‐responsive vesicles and, at the same time, resist the large compressive force exerted by the expanding lipid droplet. Insulin‐stimulated glucose uptake depends on a precise relay system. Before GLUT4 vesicles can reach the cortical actin network, they have to travel through the deep cytoplasm along microtubule tracks using kinesin motor proteins [60, 61]. Phosphoproteomic analyses have revealed that this track formation is actively regulated by insulin. Parker et al. [62] discovered that insulin regulates the phosphorylation of microtubule plus‐end tracking proteins (+TIPs) such as CLASP2, G2L1, MARK2, EB1 and CLIP2. Crucially, this study identified a novel mechanism of stabilization: upon insulin stimulation, these +TIPs redistribute from exclusively tracking the growing tip to trailing behind it. While insulin increases the number of CLASP2‐containing microtubule ends, it simultaneously decreases their velocity. The decrease in microtubule dynamics by the trailing +TIPs stabilizes the tracks to ensure efficient delivery of glucose transporters [62]. However, in hypertrophic adipocytes, this long‐range transport is compromised. The physical expansion of the giant unilocular lipid droplet severely distorts and reorients the microtubule lattice around its perimeter, creating a mechanical and spatial traffic jam that traps GLUT4 storage vesicles in the perinuclear region [63]. Even if the insulin signalling pathway (PI3K/Akt) is intact, the stable structural tracks required to deliver the glucose transporter to the plasma membrane are disrupted, and this leads to insulin resistance [60]. Such mechanisms have been observed in skeletal muscle cells, where disruptions to microtubule‐mediated GLUT4 trafficking also induced insulin resistance in spite of proper PI3K and Akt activation [64].

### 3.2 Lipid Droplet Dynamics: Fusion and Organelle Interaction

In addition to GLUT4, microtubules are the primary tracks for lipid droplet transport and fusion. Lipid droplets can increase in size not only by triglyceride biosynthesis but also by the fusion of smaller droplets into larger ones. Lipid droplets are not static; they move actively within the cell. This movement is mainly mediated by microtubule‐associated motor proteins such as kinesin and dynein that walk along microtubule tracks. This motor protein‐mediated transport is important for the fusion of smaller lipid droplets into larger ones, and this process is critical for adipocyte growth. Bostrom et al. [65] reported that the process of lipid droplet complex formation is dependent on an intact microtubule network, and this process was inhibited when microtubules were depolymerized using nocodazole. The study suggests that microtubules are required not only for the long‐distance transport of lipid droplets but also for bringing them close together so that fusion can occur.

Dynein motor proteins are involved in this microtubule‐dependent fusion of lipid droplets [66, 67]. The motor‐driven transport is also important for the lipid droplets to travel and interact with other organelles such as the endoplasmic reticulum, mitochondria and lysosomes, and these interactions are crucial for lipid metabolism, signalling and nutrient exchange [68, 69, 70]. Any disruptions in the motor protein‐driven microtubule‐based transport can contribute to metabolic diseases like obesity and fatty liver disease by impairing the necessary communication between lipid droplets and the rest of the cell. However, the authors point out that this mechanism is prominent in cells such as hepatocytes, which have smaller lipid droplets compared to white adipocytes, where most of the cytoplasm is occupied by a few large lipid droplets, and there is little space for them to move around in the cell.

### 3.3 The Lipolysis–Lipogenesis Cycle

Microtubules also play a key role in the lipolysis–lipogenesis cycle of white adipocytes. After the lipolysis process, the macro lipid droplets shrink to release the fat, and they form micro lipid droplets. In order to return to its basal lipid storage state, via an insulin‐dependent process, the micro lipid droplets fuse together to form a single large macro lipid droplet. Microtubules are required for the homotypic fusion of the micro lipid droplets [66, 67]. The microtubule‐dependent, insulin‐driven fusion of lipid droplets allows adipocytes to increase their size after lipolysis, indicating that microtubule‐dependent signalling pathways are required for maintaining lipid homoeostasis in adipocytes.

### 3.4 Microtubule Acetylation: The Stability Switch

The function of the microtubule network in hypertrophic adipocytes—whether it acts as a stable track for the transport of GLUT4 vesicles or disassembles to allow lipid droplet growth—is determined by its post‐translational modifications, specifically acetylation. Microtubule acetylation is regulated by the acetyltransferase MEC‐17 (αTAT1) and deacetylases SIRT2 and HDAC6. Insulin signalling itself can increase α‐tubulin Lysine 40 (K40) acetylation through a counterbalance between GSK3 and mTOR signalling. This insulin‐stimulated acetylation is intended to stabilise the microtubule lattice to support the +TIP ‘trailing’ phenomenon and to facilitate transport [62]. But during adipocyte hypertrophy, this acetylation signal is used to induce cytoskeletal remodelling. During adipogenesis, the microtubules remodel to allow the lipid droplet to expand. This is achieved by upregulating MEC‐17 to increase α‐tubulin acetylation. The increased acetylation acts as a signal to induce katanin‐mediated microtubule‐severing and subsequent cytoskeleton remodelling, which allows the expansion of lipid droplets and accelerates the transformation of the cell into a mature adipocyte [71]. Even though the microtubule severing allows lipid droplet expansion, it can disrupt the GLUT4 vesicle transport. Since kinesin‐1 motor proteins require long, stable acetylated microtubules to efficiently transport GLUT4 vesicles [72], the fragmentation of these tracks impairs GLUT4 translocation to the plasma membrane. Thus, metabolic dysfunction can happen when the high acetylation that is intended for insulin‐driven transport is instead used by the severing machinery to accommodate lipid droplet growth.

### 3.5 Mechanotransduction: The LINC Complex and the Microtubule‐Actin Crosstalk

Although the disassembly of the microtubule network is required to accommodate the expanding lipid droplets, microtubules also help to transmit mechanical information directly to the nucleus. Mechanotransduction in adipocytes depends on the interaction between the cytoskeleton and the nucleus. Yang et al. [73] demonstrated that the cross‐talk between microtubules and SUN2, which is a core component of the Linker of Nucleoskeleton and Cytoskeleton (LINC) complex, is essential for adipogenesis. The Microtubule‐SUN2 interaction regulates the mechanical stiffness of the adipocyte. This interaction also helps in the nuclear transport of PPARγ. Disruption of the Microtubule‐LINC interaction inhibits the recruitment of PPARγ to the nucleus, which prevents adipogenic differentiation. This suggests that the microtubule network is not just a passive scaffold, but it is also an active regulator of adipogenic gene expression.

Microtubule dynamics can directly influence actin contractility via specific signalling factors. In many cell types, microtubules sequester the Rho‐activator GEF‐H1. When microtubules undergo massive remodelling or depolymerization, which also happens during adipocyte hypertrophy, GEF‐H1 is released into the cytoplasm where it activates RhoA and triggers the formation of actin stress fibres [74]. This mechanism might be responsible for the pathological elevation of RhoA‐ROCK signalling observed in obese adipose tissue and the resulting actin stiffness [56]. Thus, the cross‐talk between actin and microtubules could increase cytoskeletal stiffness and metabolic defects in adipocytes.

## 4 Vimentin: Structural Cage and Extracellular Signal

Vimentin filaments are considered the passive skeletal support of the adipocyte cell. But recent studies suggest that the vimentin network is a critical, active regulator of lipid metabolism, regulating lipolysis, and it also acts as an extracellular signalling molecule. During adipogenesis, the vimentin intermediate filament network undergoes significant reorganization. It changes from an extended cytoplasmic network to form a distinct ‘cage‐like’ cortex around developing lipid droplets. This remodelling is essential for maintaining the mechanical integrity of the adipocyte as the lipid droplet expands [75].

### 4.1 The Vimentin–Perilipin Connection

Heid et al. [76] identified that the organization of vimentin around the lipid droplets is mediated by Perilipin‐1. They proposed a model for lipid droplet biogenesis in which Perilipin‐1 is the first protein that is recruited to the surface of nascent droplets. The Perilipin then acts as an anchor to recruit vimentin filaments, which form a cortex around the lipid droplet. The cortex helps to organize and stabilize the droplets so that the droplets can grow larger without undergoing premature fusion. The study also showed that the vimentin‐perilipin complex on the lipid droplet surface interacts with multiple layers of smooth endoplasmic reticulum. This suggests that there is a well‐organized system for lipid droplet formation, where ER is responsible for synthesizing the lipids, perilipin and vimentin organize the surface of the droplet, and the entire structure is coordinated to carry out efficient lipid storage.

### 4.2 Metabolic Regulation: Lipid Uptake and Lipolysis

In addition to its structural role, the vimentin cage also functions as a dynamic interface that regulates both lipid uptake and degradation. Vimentin regulates the entry of lipids by interacting with CD36, which is a major fatty acid translocase. Vimentin deficiency reduces the trafficking of CD36 to the plasma membrane, causing the decreased membrane localization of CD36, and hence, there will be less uptake of fatty acids. The vimentin cage also acts as a necessary scaffold for lipolysis via its interaction with Hormone‐Sensitive Lipase (HSL) [77]. HSL is the main enzyme that degrades stored fat. HSL is activated by phosphorylation, and it is then translocated from the cytosol to the lipid droplet to degrade lipids into fatty acids to produce energy. In vimentin null adipocytes, translocation of HSL to the lipid droplet is impaired [78]. Without the structural support of vimentin, HSL cannot interact effectively with the lipid droplet to catalyse lipid breakdown, indicating that vimentin is important for lipolysis.

### 4.3 The Extracellular Vimentin as Signalling Molecule

**Figure 2:**
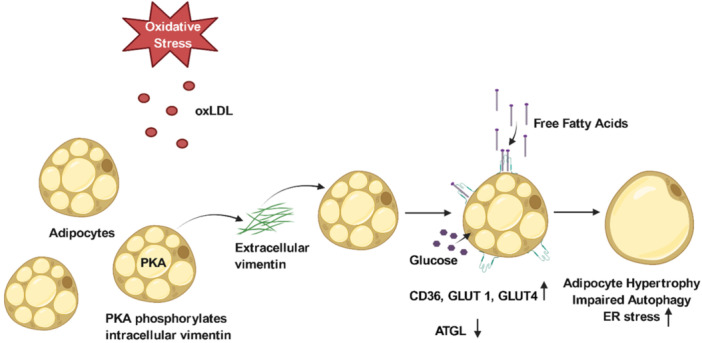
Extracellular vimentin facilitates increased free fatty acid uptake in adipocytes under oxidative stress. Adipocytes exposed to oxidized low‐density lipoprotein (oxLDL) experience oxidative stress, which activates Protein Kinase A (PKA). PKA phosphorylates intracellular vimentin triggering its secretion into the extracellular space. The extracellular vimentin functions as a signalling molecule that enhances the cell surface expression of CD36, GLUT1 and GLUT4 and decreased the expression of lipase ATGL. This resulted in the increased uptake of free fatty acids and glucose. The nutrient overload, together with impaired autophagy and increased ER stress facilitates the expansion of intracellular lipid droplets and the transition to a dysfunctional, unilocular hypertrophic phenotype. Here, the multilocular cells represent the intermediate phase of rapid lipid uptake that precedes complete droplet fusion into a unilocular hypertrophic white adipocyte. Created in BioRender. https://BioRender.com/13trpxl.

A recent study revealed that 3T3L1‐derived adipocytes secrete vimentin in response to oxidized low‐density lipoprotein (oxLDL), and the extracellular vimentin acts as a stress‐induced signal that modifies adipocytes towards a hypertrophic and pro‐inflammatory state [79] (Figure 2). When the oxidized low‐density lipoprotein binds to the CD36 receptor, protein kinase A is activated, which phosphorylates vimentin and triggers its secretion into the extracellular space. When adipocytes are exposed to the extracellular vimentin, it causes an increase in the size of intracellular lipid droplets and increases the levels of intracellular triglycerides, directly promoting adipocyte hypertrophy. Extracellular vimentin increased energy intake by upregulating the expression of both the fatty acid transporter CD36 and glucose transporters (GLUT1 and GLUT4) on the plasma membrane. It promotes lipid accumulation not only by increasing lipid intake but also by suppressing lipid breakdown by downregulating key lipolytic enzymes like ATGL (Adipose Triglyceride Lipase). Extracellular vimentin also inhibits autophagy and increases ER stress, which also contributes to the obesity related metabolic dysfunctions. Here, vimentin acts as a secreted adipokine rather than merely as an internal support structure. By enhancing CD36‐dependent lipid accumulation and by inhibiting lipolysis, extracellular vimentin acts as a direct promoter of adipocyte hypertrophy. Future studies must address the underlying mechanism of this extracellular signalling to determine if targeting extracellular vimentin can regulate the inflammation‐obesity crosstalk and also determine whether other cytoskeletal proteins are similarly secreted to regulate adipose tissue function and metabolic health.

### 4.4 Vimentin and GLUT4 Retention

Vimentin also plays a specific role in insulin resistance, which is distinct from the functions of actin and microtubules. In the basal unstimulated state, vimentin retains GLUT4 storage vesicles in the perinuclear region, preventing their premature release. The vimentin network acts as a scaffold that holds the GLUT4 storage vesicles in a clustered, ready‐to‐go state near the cell centre, so that they can be released in response to insulin signalling. Vimentin directly binds to insulin‐responsive aminopeptidase (IRAP), a protein that travels with GLUT4 on the storage vesicles. When vimentin was depleted from adipocytes, the GLUT4 vesicles were found to be dispersed, and their insulin‐stimulated movement to the cell surface was reduced [80]. Adipocytes from vimentin‐depleted mice had less plasma membrane expression of GLUT4, leading to reduced glucose uptake, which further indicates that vimentin is crucial for the intracellular trafficking of GLUT4 in adipocytes [78]. In hypertrophic adipocytes, the physical distortion of the vimentin cage by the expanding lipid droplet can disrupt the retention of GLUT4 at the cell centre. This leads to either the degradation of GLUT4 vesicles or defects in their sorting, resulting in the reduction in insulin‐responsive glucose uptake observed in obesity.

## 5 Septins: The “Fourth Component” of Cytoskeleton

Septins (SEPTs) are a highly conserved family of GTP‐binding proteins that are now considered the “fourth component” of the cytoskeleton. Even though the functions of actin filaments, microtubules and intermediate filaments in adipocytes are well established, the function of SEPTs remains largely unexplored and complex. SEPTs are divided into four subgroups (SEPT2, SEPT6, SEPT7 and SEPT3), and they assemble into hetero‐oligomeric complexes which further organize into higher‐order structures such as filaments, rings, bundles and cage‐like structures. The SEPT structures localize to various regions within the cell, such as the plasma membrane, the cleavage furrow during cytokinesis, and on the organelle surfaces. SEPT isoforms ‐ SEPT7 and SEPT11 ‐ have been associated with adipogenesis and lipid droplet formation [81, 82, 83]. Studies carried out in non‐adipocyte cells, such as liver cells and Hepatitis C viral infection, suggest that SEPT9 influences the perinuclear clustering and trafficking of lipid droplets, but its direct role in adipogenesis or hypertrophy is not established [84, 85, 86]. In addition to regulating lipid dynamics, SEPTs also regulate vesicle trafficking. By coordinating the transport machinery, they also influence the translocation of GLUT4 vesicles [87].

SEPTs might play a key mechanobiological role during hypertrophy, when the cells undergo large physical expansion. They function along with actin filaments, microtubules and intermediate filaments to regulate membrane dynamics, cortical rigidity and vesicle trafficking [88, 89, 90, 91, 92, 93]. During adipocyte hypertrophy, when extensive cytoskeletal remodelling occurs, SEPTs are hypothesized to act as mechanical scaffolds that stabilize the plasma membrane against increasing tension and help to maintain cellular integrity, preventing cell rupture. Further, SEPTs may also function as scaffolds controlling the fusion and fission of lipid droplets, which is essential for lipid storage and expansion.

### 5.1 SEPT11: Scaffold for Loading Lipids

Among the mammalian SEPT isoforms, SEPT11, which is a member of the SEPT6 subgroup, acts as a critical link between the cytoskeletal architecture and metabolic function. SEPT11 is associated with cytokinesis, cell migration, vesicle trafficking, haematopoiesis, tumour progression and neurodegeneration [94, 95, 96]. Recent studies carried out in human adipocytes suggest that SEPT11 is a central player in obesity, lipid traffic and the regulation of lipid metabolism [82].

SEPT11 expression is upregulated in the adipose tissue of obese individuals, where it correlates positively with adiposity markers in omental fat and with insulin resistance in subcutaneous fat. The regulation of SEPT11 is related to adipocyte size rather than to insulin resistance. When mouse 3T3‐L1 cells and human adipocytes were exposed to lipogenic agents like oleate and insulin, there was an increase in the level of SEPT11 protein. As the adipocytes expanded, SEPT11 underwent extensive reorganization from long linear bundles to sub‐membranous structures. This rearrangement was similar to the actin cytoskeleton remodelling that occurred during adipogenesis, suggesting a functional interaction between SEPT11 and actin. SEPT11 is assembled into either filaments or rings, depending on the stage of adipocyte differentiation and its interaction with other SEPTs such as SEPT2 or SEPT9 [82].

### 5.2 Coordination of Lipid Trafficking and Insulin Signalling

SEPT11 functions as a dynamic scaffold for lipid transport machinery in adipocytes. It is associated with Caveolin‐1 in the adipocyte caveolae and also with Fatty Acid‐Binding Protein 5 (FABP5). When adipocytes were loaded with lipids, the three proteins ‐ SEPT11, caveolin‐1, and FABP5 moved to the surface of the lipid droplets, and they worked together to facilitate lipid loading and transport. This scaffolding function of SEPT11 is critical because the siRNA‐mediated silencing of SEPT11 significantly reduced the levels of both Caveolin‐1 and FABP5, destabilizing the lipid uptake machinery, but there was no effect on other adipogenic markers [82].

Beyond its structural role, SEPT11 is essential for maintaining insulin signalling in adipocytes. Silencing of SEPT11 in 3T3‐L1 adipocytes reduced insulin‐stimulated Akt phosphorylation, indicating impaired insulin signalling. Depletion of SEPT11 also decreased basal and insulin‐induced lipogenesis, intracellular lipid accumulation, and suppressed lipolytic rates. These findings indicate that silencing SEPT11 impairs insulin signalling and the ability of adipocytes to accumulate lipids in response to insulin [82].

### 5.3 The SEPT7 Paradox: Scaffold or Brake?

Although SEPT11 appears to support adipocyte expansion, the function of SEPT7 is controversial. SEPT7 is the core monomer within the SEPT complexes, and it attaches to the ends of hexameric building blocks to form non‐polarized filaments. As the central subunit required for the stability of most SEPT filaments, SEPT7 shows two opposing functions in adipocyte biology: it acts as a scaffold for lipid droplet biosynthesis and also a mechanical brake against uncontrolled hypertrophy.

### 5.4 The Pro‐Adipogenic Function of SEPT7

Early studies by Cheng et al. [81] and Chen et al. [39] suggest that SEPT7 is an essential driver of adipogenesis. In 3T3‐L1 and bovine pre‐adipocytes, SEPT7 expression increases during differentiation, and it coordinates the transition from fibroblast‐like precursor cells to mature fat cells. Overexpression of SEPT7 increases lipid droplet accumulation and promotes adipogenic differentiation, whereas silencing SEPT7 reduces lipid deposition. SEPT7 expression is also associated with the upregulation of PPARγ and CEBPα genes, which are the master regulators of adipogenesis and lipogenesis. SEPT7 is crucial for maintaining MEK/ERK pathway activity. Since ERK1/2 phosphorylation is required for the transcriptional activation of the master regulators PPARγ and CEBPα, SEPT7 depletion prevents adipogenic differentiation. Other than upregulating the lipogenic genes, SEPT7 promotes lipid storage by downregulating the expression of adipose triglyceride lipase, which is the rate‐limiting enzyme of triglyceride hydrolysis [81].

In addition to its transcriptional function, SEPT7 also has a critical structural function during lipid droplet biogenesis. Compared to other SEPT family members such as SEPT2, SEPT6 and SEPT9, SEPT7 binds strongly to FIT2 (Fat Storage‐Inducing Transmembrane Protein 2). SEPT7 is recruited by the cytosolic loops of FIT2 to the ER‐lipid droplet junction, where it acts as a cytoskeletal scaffold. Together with ER tubule‐forming proteins, this complex generates the membrane curvature required to initiate lipid droplet biogenesis. This process was identified not only in *C. elegans* but also in non‐adipocyte cells such as HepG2 and COS‐7 cells, indicating that it is an evolutionarily conserved mechanism. Depletion of SEPT7 reduced the number and size of lipid droplets, confirming that SEPT7 is crucial for the synthesis of lipid droplets. Although these studies were conducted in non‐adipose cells, the significant upregulation of FIT2 and SEPT7 in 3T3L1 adipocytes indicates that adipocytes might also use the same mechanism for proper fat storage [97]. Thus, by modulating adipogenesis at the transcriptional level and helping to form the structural machinery required for lipid droplet biogenesis, SEPT7 ensures the proper maturation and lipid accumulation in adipocytes.

### 5.5 The Anti‐Obesity Function of SEPT7

However, a study by Xu et al. [83] has identified SEPT7 as a negative regulator of lipid accumulation, a finding that challenges the pro‐adipogenic functions of SEPT7. Using single‐cell mapping and adipocyte‐specific SEPT7 knockout mice, the study identified SEPT7 as a negative regulator of lipid accumulation and suggests that SEPT7 has an anti‐obesity function. Mice lacking SEPT7 in adipocytes promoted white adipocyte hypertrophy and increased adipocyte number after prolonged high‐fat diet feeding, leading to obesity, reduced glucose tolerance, hepatic lipid accumulation and elevated serum triglycerides. This suggests that without SEPT7, adipocytes lose their mechanical constraint and expand excessively. This indicates that the normal function of SEPT7 is to prevent adipocyte expansion.

The study identified a signalling pathway where SEPT7 cortex inhibits Store‐Operated Calcium Entry (SOCE). By restricting SOCE, SEPT7 prevents excessive calcium influx into the cell. The restriction of intracellular calcium suppresses the expression of adipogenic master regulators (PPARγ and C/EBPα), whereas it promotes the expression of hormone‐sensitive lipase to increase lipolysis. Further, SEPT7 also regulates lipogenesis by suppressing the expression of genes involved in triglyceride synthesis (Scd1, Dgat1, Dgat2), lipid uptake (Cd36, Glut4, FABP4), and lipid droplet stability (Perilipin). Thus, by limiting the calcium signals required for lipid storage, SEPT7 prevents the adipocyte from entering a pathological hypertrophic state. Thus, SEPT7 acts as a “molecular brake” that physically and biochemically prevents the adipocyte from expanding beyond a healthy limit.

### 5.6 Context‐Dependent Function of SEPT7: Differentiation Versus Maintenance

**Figure 3:**
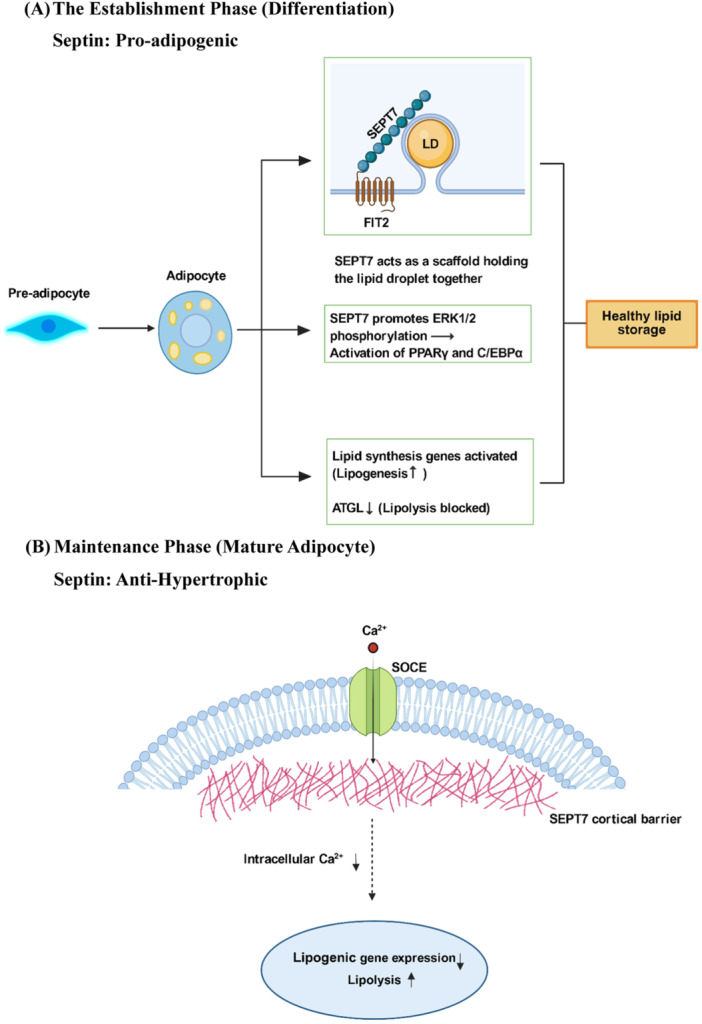
The context‐dependent Function of SEPT7 in adipocyte biology. SEPT7 functions as a scaffold during adipogenesis and a molecular brake in mature white adipocytes. (A) The Establishment Phase (Differentiation): During the transition from pre‐adipocyte to adipocyte, SEPT7 acts as a pro‐adipogenic factor. This early developmental stage is characterized by a multilocular phenotype, where the cell has numerous small, distinct lipid droplets. At the ER‐lipid droplet junction, SEPT7 is recruited by FIT2 to provide the necessary membrane curvature and cytoskeletal scaffolding needed for lipid droplet biogenesis. SEPT7 also supports the MEK/ERK signalling; phosphorylated ERK1/2 triggers the transcriptional activation of the master regulators PPARγ and C/EBPα. This signalling cascade promotes adipogenesis by activating genes associated with lipid synthesis and suppressing ATGL‐mediated lipolysis, ensuring healthy lipid storage and cellular maturation. (B) The Maintenance Phase (Mature Adipocyte): In the fully differentiated state, SEPT7 functions as a molecular brake to prevent pathological hypertrophy. SEPT7 filaments organize into a cortical barrier beneath the plasma membrane to restrict Store‐Operated Calcium Entry (SOCE). The decrease in intracellular calcium acts as a feedback mechanism to suppress the expression of lipogenic genes and promotes lipolysis by activating HSL. This prevents the adipocyte from expanding beyond mechanical limits, thereby protecting against systemic obesity and metabolic dysfunction. Under high‐fat diet conditions, SEPT7 upregulation may represent a compensatory attempt to stabilize the cell against hypertrophy. Loss of SEPT7 leads to increased calcium influx and obesity. Created in BioRender. https://biorender.com/g4efvox; https://biorender.com/vaa2647.

The contrasting findings of Cheng et al. [81], suggesting SEPT7 promotes obesity, and the findings of Xu et al. [83], suggesting that it prevents obesity, could be resolved by looking at how SEPT7 functions during the different life stages of the adipocyte. We propose that SEPT7 may function differently during adipocyte differentiation compared to the maintenance of a mature fat cell. During the initial differentiation of a pre‐adipocyte (Establishment phase), SEPT7 is required to support the ERK signalling pathway, which activates the key genes PPARγ and CEBPα and together with FIT2, it functions as a scaffold to build the initial structure of the lipid droplet. Without SEPT7, the cell cannot differentiate or build the machinery for lipid storage. Once the cell is fully formed and mature (Maintenance Phase), SEPT7 might act as a stabilizer or regulator. In a mature adipocyte, SEPT7 restricts Store‐Operated Calcium Entry. By limiting calcium influx, it prevents the lipid storage capability of the cell and encourages lipolysis (Figure 3). Thus, SEPT7 stops the cell from expanding beyond a healthy limit. The observation that SEPT7 expression is elevated in individuals with higher BMI and in mice fed a high‐fat diet [83] might represent a compensatory mechanism. Under high‐fat diet conditions, when the adipocytes are forced to expand, the cells might upregulate SEPT7 as an attempt to maintain structural integrity and metabolic function against the mechanical stress induced by hypertrophy.

### 5.7 SEPTs: A Potential Barrier for Glucose Transport

Even though the role of SEPTs in glucose transport has not been investigated in adipocytes, studies carried out in podocyte cells, which are also an insulin‐sensitive cell, suggest that SEPT7 can act as a negative regulator of glucose transport. In podocytes, SEPT7 forms complexes that suppress the activity of myosin IIA motor protein, which is essential for the final docking and fusion of GLUT4 vesicles with the plasma membrane. SEPT7 might act as a barrier to vesicle fusion. SEPT7 knockdown was found to increase the interaction between the vesicle‐SNARE VAMP2 and the plasma membrane proteins Syntaxin‐4 and Nephrin [87, 98]. The regulation of SEPT 7 on glucose transport is PI3K pathway‐independent. This implies that SEPT7 controls the physical trafficking events downstream of the insulin receptor signalling cascade. Therefore, for efficient glucose uptake, the SEPT barrier must be removed. Based on these findings, a similar mechanism might also occur in adipocytes. Therefore, the upregulation of SEPT7 observed in obesity could block effective GLUT4 trafficking. Even if insulin signalling remains intact, increased SEPT7 at the cortex could physically block myosin IIA function and shield the SNARE complex, preventing GLUT4 vesicles from fusing with the plasma membrane. This suggests that the SEPT cytoskeleton might have a crucial role in glucose uptake, and disruption of the normal function of SEPT7 in adipocytes could lead to insulin resistance and related metabolic disorders.

As discussed in the above sections, the adipocyte cytoskeleton is not merely a static scaffold but a dynamic regulator of cellular fate. Each component of the cytoskeleton actin, microtubules, vimentin, and SEPTs has its own distinct functions in adipocytes, but they function in a highly coordinated manner to perform the structural and metabolic activities in adipocytes. Obesity‐induced insulin resistance has traditionally been studied as defects in the signalling pathways‐ specifically, the downregulation of the Insulin Receptor substrate (IRS‐1) and PI3K/Akt pathway. But the signalling pathways cannot be viewed in isolation from the structural machinery that is required to translate these signals into physiological action. Insulin resistance in hypertrophic adipocytes can also occur due to the failure of the cytoskeletal trafficking machinery. Even if the upstream PI3K/Akt signal is successfully received, the pathway required to deliver the glucose transporter (GLUT4) to the cell surface can be disrupted, leading to insulin resistance. Previous studies have identified defects in cytoskeletal remodelling and stability in adipocyte hypertrophy‐associated metabolic defects [99]. To provide a clear overview of how the different cytoskeletal elements coordinate the complex life‐cycle of adipocytes, from early differentiation to the metabolic dysregulation observed in hypertrophy, we have summarized the molecular mechanisms and functional consequences in Table 1.

**Table 1:** Cytoskeletal regulation of adipocyte physiology and metabolism.

Cellular process	Cytoskeletal component	Molecular mechanism	Functional outcome	References
Adipocyte differentiation	Actin	Actin‐regulating proteins such as Cotl1 regulate actin depolymerization and PPARγ expression.	Essential for the transition from fibroblast shape to adipocyte. Absence of Cotl1 prevents differentiation.	[40]
	Microtubules	Interaction with SUN2 (LINC complex) recruits PPARγ to the nucleus.	Regulates nuclear transport of PPARγ and expression of genes required for adipogenesis.	[73]
	SEPTs (SEPT7)	Supports MEK/ERK signalling pathway	Required for transcriptional activation of master regulators PPARγ and C/EBPα	[81]
Lipid droplet formation	SEPTs (SEPT7)	Interaction with FIT2 at ER‐lipid droplet junctions	Produces membrane curvature needed to initiate lipid droplet budding/formation	[97]
	Vimentin	Recruited by Perilipin‐1 to nascent droplets	Forms the initial protective cortex around new droplets	[76]
Lipogenesis (Lipid Uptake)	SEPTs (SEPT11)	Scaffolds Caveolin‐1 and FABP5 at the droplet surface	Facilitates efficient loading and transport of lipids into the droplet	[82]
	Vimentin	Extracellular vimentin acts as a ligand signalling via CD36	Increases fatty acid uptake and promotes intracellular triglyceride accumulation	[77]
Lipid droplet expansion	Microtubules	Acts as a track for Dynein/Kinesin motor proteins	Helps in the homotypic fusion of lipid droplets (fusion of smaller lipid droplets to form larger droplets)	[66]
	Actin	Polymerization into stress fibres via the RhoA/ROCK pathway	Provides tensile strength to prevent cell rupture during the excessive increase in cell volume	[48]
Lipolysis	Vimentin	Forms a cage that scaffolds Hormone‐Sensitive Lipase (HSL)	Essential for the docking of HSL. Loss of vimentin impairs lipolysis	[77]
	SEPTs (SEPT7)	Inhibits Store‐Operated Ca2+ Entry (SOCE)	Low intracellular Ca2+ promotes lipolysis and suppresses lipogenic genes	[83]
Hypertrophy & Mechanotrans‐ duction	Actin/YAP/TAZ	Mechanical stretch stiffens actin → YAP/TAZ enters the nucleus	Repression: Blocks PPARγ (stops lipid storage) Activation: Upregulates Leptin (uncouples leptin expression from fat mass & preserves systemic insulin sensitivity in spite of lipid storage defects	[49, 50, 51, 52]
	Microtubules	Deacetylation of microtubules by SIRT2 and severing by Katanin	Destabilizes microtubule network to allow physical space for droplet expansion, but this leads to a traffic jam in the transport highway	[71]
Metabolic Function (Glucose Transport)	Vimentin	Retains GLUT4 vesicles in the perinuclear region	Distortion of the vimentin cage traps GLUT4 vesicles, preventing their insulin‐responsive release	[78]
	Microtubules	Long‐range transport tracks of GLUT4 vesicles carried by kinesin motor proteins	Disassembled/blocked tracks prevent GLUT4 from reaching the cell cortex	[60, 61]
	Actin	Cortical network for the docking of GLUT4 vesicles with the plasma membrane	Stiff cortical actin (creates a physical barrier to GLUT4 fusion)	[41]
	SEPTs	Forms a cortical barrier that might block myosin IIA motor proteins	Physical shielding of SNARE complexes prevent final glucose uptake	[87, 98]

## 6 Conclusion and Future Perspectives

The transition from a healthy, insulin‐sensitive adipocyte to a hypertrophic, insulin‐resistant state is not just a metabolic event, but it also involves multi‐stage mechanical failure of the cytoskeleton. This review highlights the cytoskeleton as the central integrator of this transformation. It starts with the actin network acting as a primary mechanosensor at the cell surface, converting external fibrotic ECM tension and internal cell expansion forces into gene expression‐regulating signals via the RhoA‐ROCK‐YAP/TAZ pathways. As hypertrophy progresses, the microtubule network, which is the cell's primary traffic route, is physically compromised, resulting in a traffic jam of GLUT4 vesicles that prevents insulin‐stimulated glucose uptake. Simultaneously, the vimentin cage must adapt to provide structural reinforcement, while the Septin family acts as a molecular brake to adjust the rate of lipid accumulation. When these physical tracks and docking stations are structurally compromised by over‐expansion, even a perfectly executed upstream biochemical signal cannot translate into proper metabolic action.

By recognizing the cytoskeleton as a coordinated mechanical machine rather than as a collection of passive scaffolds, we suggest that targeting the mechanical properties of the adipocyte could act as a novel therapeutic strategy to address the root causes of cellular dysfunction rather than merely treating the metabolic symptoms. Transitioning our understanding of obesity‐induced insulin resistance from a purely metabolic signalling defect to a disease of cellular mechanics opens entirely new avenues for intervention.

To advance research in obesity and adipocyte biology, future studies should aim to restore the mechanical equilibrium of the tissue by prioritizing three major areas:

a. **Resolving the function of Septin:** The dual function of SEPT7 (pro‐adipogenic vs. anti‐obesity) must be clarified using inducible, stage‐specific knockout models. This will determine whether SEPT7 can be pharmacologically regulated to allow the healthy expansion of adipocytes without triggering pathological hypertrophy.
b. **Exploring the cytoskeletal secretome:** The discovery of extracellular vimentin suggests that a comprehensive proteomic analysis of the cytoskeletal secretome is required to determine whether other structural proteins can get secreted and function as pro‐inflammatory adipokines.
c. **Targeting adipocyte mechanics for therapy:** Future strategies must investigate whether modulating cell stiffness can successfully uncouple obesity from metabolic disease. Utilizing ROCK inhibitors or agents that target actin dynamics will be critical to evaluate whether decreasing the cellular stiffness of hypertrophic adipocytes can restore insulin sensitivity, even in the chronic context of obesity.

Therefore, reframing obesity as a failure of cytoskeletal mechanics allows us to target the physical root cause of the condition, ensuring that the adipocyte retains its vital metabolic functions even during periods of cellular expansion.

## Author Contributions

R.M. conceived the idea and topic. D.S. and R.M. conceptualised and designed the systematic review. Both authors contributed equally to the literature search and data curation. R.M. and D.S. have read, edited, and approved the final version of the manuscript to be published.

## Funding

The authors have nothing to report.

## Conflicts of Interest

The authors declare no conflicts of interest.

## Declaration of Generative AI Use

During the preparation of this review article in February 2026, the authors used Gemini 2.0 in order to rephrase the sentences strictly for refining language in the Introduction and Conclusion and Future Perspectives sections. After using this tool, the authors critically reviewed, verified and edited the text to ensure scientific accuracy and take full responsibility for the content of the published article.

## Data Availability

Data sharing not applicable to this article as no datasets were generated or analysed during the current study.
